# Progression and Augmentation Therapy in PiSZ and PiZZ Alpha-1 Antitrypsin Deficiency: A Longitudinal Functional and Densitometric Study

**DOI:** 10.3390/biom15040599

**Published:** 2025-04-17

**Authors:** Soha Esmaili, Juan Luis Rodríguez Hermosa, Gianna Vargas Centanaro, José Luis Álvarez-Sala, Iman Esmaili, Myriam Calle Rubio

**Affiliations:** 1 Pulmonology Department, Hospital Clínico San Carlos, 28040 Madrid, Spain; soha@esmaili.ws (S.E.); gvargas@alumni.unav.es (G.V.C.); jlasw@separ.es (J.L.Á.-S.); mcalllerubio@gmail.com (M.C.R.); 2 Heart Lung Innovation Centre, Vancouver, BC V6Z 1Y6, Canada; 3 Instituto de Investigación Sanitaria del Hospital Clínico San Carlos (IdISSC), 28040 Madrid, Spain; 4 Department of Medicine, School of Medicine, Universidad Complutense de Madrid, 28040 Madrid, Spain; 5 ISNS Data Analytics and Research, Vancouver, BC V6T 1Z3, Canada; iman@esmaili.ca; 6 CIBER de Enfermedades Respiratorias (CIBERES), 28029 Madrid, Spain

**Keywords:** Alpha-1 antitrypsin deficiency, PiSZ genotype, PiZZ genotype, augmentation therapy, lung densitometry

## Abstract

Background: Alpha-1 antitrypsin deficiency (AATD) is a genetic disorder associated with an increased risk of developing chronic obstructive pulmonary disease (COPD) with variable phenotypic expression among different genotypes. While the PiZZ genotype is well characterized, the clinical and structural progression of PiSZ individuals remains less defined. This study evaluates genotype-specific disease trajectories and the impact of augmentation therapy over a two-year follow-up. Methods: A prospective observational cohort study was conducted, including 74 AATD patients (41 PiSZ, 33 PiZZ), stratified by augmentation therapy status. Disease progression was assessed through lung function decline (forced expiratory volume in one second [FEV1], diffusing capacity for carbon monoxide [DLCO], carbon monoxide transfer coefficient [KCO]) and densitometric changes (15th percentile lung density [PD-15], percentage of lung voxels below −950 Hounsfield units [HU-950]). Mixed-effects models and multivariable regression analyses were performed to evaluate genotype-specific progression patterns and treatment effects. Results: Results: PiZZ individuals exhibited significantly greater annual decline in lung function and densitometric parameters compared to PiSZ individuals, with more pronounced loss in basal lung regions and with greater decline in advanced stages, in contrast to the PiSZ genotype, which showed greater progression in earlier stages. Augmentation therapy was associated with a significant reduction in PD-15 decline in both genotypes, with the greatest benefit observed in PiZZ patients and in those diagnosed within five years of disease onset. Smoking and frequent exacerbations were identified as independent risk factors for accelerated disease progression. Conclusions: PiZZ individuals experience a more aggressive disease trajectory than PiSZ individuals in the absence of treatment. Augmentation therapy effectively mitigates disease progression in both genotypes, with greater efficacy when initiated early. Smoking and frequent exacerbations were identified as independent risk factors for accelerated disease progression. These findings underscore the importance of genotype-specific monitoring and personalized therapeutic strategies in AATD to optimize clinical outcomes.

## 1. Introduction

Alpha-1 antitrypsin deficiency (AATD) is a hereditary disorder characterized by reduced circulating levels of alpha-1 antitrypsin (AAT), a major serine protease inhibitor that protects lung parenchyma from proteolytic damage. It is a well-established risk factor for the development of early-onset emphysema, particularly in individuals carrying the PiZZ genotype, which is associated with markedly severe reductions in AAT levels [1]. Despite increased awareness, AATD remains largely underdiagnosed, with many patients identified only after irreversible lung function impairment has occurred [2]. Although the PiZZ genotype is traditionally considered the primary pathogenic variant, accumulating evidence suggests that the PiSZ genotype, despite having higher residual AAT levels, may also predispose individuals to clinically significant disease [3].

A major challenge in AATD management is the substantial delay in diagnosis, often occurring only when symptoms manifest, leading to missed opportunities for early intervention [4]. While the natural history of PiZZ patients is well described, the trajectory of lung function decline in PiSZ individuals remains incompletely characterized [5]. PiSZ individuals demonstrate marked clinical heterogeneity, with some showing minimal disease progression, while others develop emphysema at rates comparable to PiZZ patients [6]. The need for refined risk stratification in PiSZ individuals is underscored by the growing recognition that a subset of these patients, particularly those with smoking exposure or recurrent exacerbations, may exhibit a rapid rate of disease progression [7]. Our prior work provided the first detailed longitudinal analysis of untreated PiSZ individuals, identifying key factors influencing disease progression in this subgroup [8]. However, the role of augmentation therapy in modifying the disease course in PiSZ remains undefined, raising important clinical and therapeutic questions.

The assessment of disease progression in AATD has traditionally relied on spirometric decline, particularly forced expiratory volume in one second (FEV1) decline, but emerging evidence suggests that computed tomography (CT) densitometry offers a more sensitive metric for detecting early lung parenchymal destruction [9,10]. Despite this, most clinical guidelines continue to focus on global lung function parameters, overlooking the potential utility of regional lung density assessment as a mechanistic predictor of decline [11,12]. While global densitometric loss correlates with functional impairment, the specific impact of regional lung density changes, particularly in the basal regions where emphysematous destruction is most pronounced, remains underexplored [12].

The spatial distribution of lung tissue loss may have significant implications for disease heterogeneity and treatment response. PiZZ individuals typically exhibit a basal-dominant pattern of emphysema, which is associated with accelerated lung function decline [13]. In contrast, PiSZ patients display a more variable densitometric profile, with some individuals exhibiting regional patterns indistinguishable from PiZZ patients, while others show a milder disease presentation [14]. Identifying whether regional densitometric loss serves as a predictor of functional impairment, particularly in PiSZ individuals, is critical for advancing AATD risk stratification [9]. Our previous findings suggested that PiSZ individuals with higher rates of basal lung density loss experienced a more rapid decline in lung function, further supporting the hypothesis that structural vulnerability in specific lung regions may be a key determinant of progression [8,15].

Augmentation therapy is the only disease-specific treatment available for AATD and has been shown to slow lung density decline in PiZZ individuals, with evidence from randomized trials such as RAPID and RAPID-OLE confirming its long-term efficacy [16]. However, its role in PiSZ individuals remains incompletely defined, with no robust evidence guiding treatment decisions in this genotype [17]. Given that a subset of PiSZ patients exhibits a progression rate comparable to PiZZ individuals, it remains uncertain whether augmentation therapy could provide meaningful clinical benefits in high-risk PiSZ patients [18]. The current study aims to address this gap by identifying PiSZ patients with rapid densitometric progression and functional decline, who may represent an overlooked target population for augmentation therapy [19].

Beyond genetic predisposition, multiple factors influence disease progression in AATD. Smoking has been shown to accelerate both functional decline and lung density loss, particularly in individuals with lower baseline FEV1 [20]. Exacerbations are another major determinant of accelerated decline, with recurrent episodes leading to irreversible lung damage and worsening long-term prognosis [21,22]. The interplay between genotype, smoking, exacerbation frequency, and time since diagnosis suggests that a multifactorial model is required to accurately predict disease trajectory [23,24].

Several models have been proposed to quantify disease progression in AATD, integrating clinical, functional, and imaging markers to improve risk stratification [25]. Prior studies have demonstrated that densitometric parameters enhance the assessment of emphysema progression and therapeutic response [26]. However, the predictive value of regional lung density loss in individualized risk assessment remains largely unexplored [27,28]. Incorporating structural imaging biomarkers into clinical models may refine patient classification, allowing for improved early detection of rapid progressors and more effective treatment allocation [29].

This study evaluates pulmonary disease trajectories and the impact of augmentation therapy on pulmonary disease progression in PiSZ and PiZZ individuals during a two-year follow-up, integrating functional and densitometric parameters while assessing the role of clinical risk factors in disease evolution. Additionally, the study aimed to analyze regional lung density loss patterns and develop predictive models of disease progression and treatment response, incorporating functional and densitometric parameters to refine risk stratification.

## 2. Materials and Methods

The methodology has been previously reported (8). Briefly, this study is a prospective observational cohort with a 2-year follow-up period, conducted between 1 March 2021 and 1 March 2023. The study included patients with diagnosis of COPD based on a post-bronchodilator FEV_1_/FVC ratio of less than 0.7 and diagnosed with AATD carrying the SZ or ZZ genotype, recruited through consecutive sampling. Exclusion criteria included the presence of uncontrolled comorbidities that could interfere with disease progression, such as active neoplasms or other chronic respiratory disorders. Participants were followed for two years, including annual office visits and a telephone follow-up call every six months to record exacerbations treated with oral corticosteroids or antibiotics or hospitalization events. Clinical variables, lung function, and chest CT (lung densitometry) were assessed at baseline and annually. Augmentation therapy was evaluated in terms of treatment status, dose, and frequency of administration.

All pulmonary function tests were conducted using standardized protocols, ensuring reproducibility and minimizing measurement variability. Multislice spiral CT scans were obtained using a standardized protocol, with images acquired at full inspiration to minimize variability due to lung volume changes. The acquisition parameters included a slice thickness of ≤1 mm, 120 kVp, and automated tube current modulation to ensure optimal image quality. All scans were performed with volumetric reconstruction to allow for densitometric analysis. The analysis was conducted using validated software for quantitative densitometry (StratX, VIDA Diagnostics, IA, USA), with lung segmentation performed automatically and corrected for inspiratory variability through a density-matching model to standardize lung volume. Lung density was quantified using PD-15 (15th percentile lung density) and HU-950 (percentage of lung volume below −950 Hounsfield units). To ensure the accuracy of densitometric measurements, all imaging data were reviewed by experienced radiologists blinded to clinical variables and disease duration, ensuring standardization in image interpretation. Additionally, pulmonary function and imaging assessments were performed within a maximum interval of 72 h to ensure comparability between functional and radiological measurements

Disease progression risk factors included time since diagnosis, smoking exposure quantified in pack-years, and the interaction between smoking and exacerbations. Patients were stratified according to time since diagnosis, categorized as either less than five years or five or more years. Patients receiving augmentation therapy were included in the treated group, while untreated patients were those who had never received augmentation therapy before or during the study. Exacerbation history was documented as the number of moderate-to-severe exacerbations per year, stratified as fewer than two or at least two exacerbations annually.

The study was approved by the Ethics Committee of the Clinical Hospital of San Carlos (CI: 18/357-E) and conducted in accordance with the Declaration of Helsinki and Spain’s Organic Law 3/2018 on data protection. Written informed consent was obtained from all participants prior to study enrollment.

### Statistical Analysis

Baseline characteristics were compared using independent t-tests for continuous variables and chi-square or Fisher’s exact tests for categorical variables. The normality of continuous variables was assessed using the Shapiro–Wilk test prior to applying parametric tests. If non-normality was detected, non-parametric alternatives such as the Mann–Whitney U test were employed. Type I error was controlled using Holm–Bonferroni corrections, while Type II error was minimized by ensuring sufficient statistical power through post hoc analysis.

Functional and densitometric progression was assessed using linear mixed models (LMM) incorporating fixed effects for genotype, treatment, smoking status, exacerbations, and time since diagnosis. Baseline FEV_1_ (% predicted) was included as a covariate in all models to account for its potential confounding effect on disease progression. Interaction terms, including genotype × treatment, time since diagnosis × treatment, and smoking × exacerbations, were evaluated to explore modifying effects on disease progression.

Analysis of covariance (ANCOVA) models were employed to adjust for potential confounders, including age, smoking pack-years, exacerbation frequency, and baseline FEV_1_ (%), ensuring that differences in initial lung function did not bias progression analyses. Holm–Bonferroni corrections were applied for multiple comparisons. All comparisons and estimations were reported with 95% confidence intervals to ensure statistical robustness and interpretability.

Densitometric progression (PD-15 loss and HU-950 changes) was analyzed using Welch’s *t*-tests, which accounted for potential heterogeneity in variance across groups, confirmed using Levene’s test. Multivariable regression models were constructed to assess predictors of regional lung density loss, stratifying patients by genotype and treatment status. Variance inflation factors (VIF) were calculated to assess multicollinearity among predictors, with a threshold of ≤5 confirming the absence of significant collinearity.

Residual diagnostics, including normality assessment and graphical evaluation of standardized residuals, were conducted to ensure model adequacy. Additional exploratory models tested the impact of exacerbation severity and disease burden on functional and densitometric decline. The classification of densitometric progression groups was validated through ANOVA, and additional bootstrap resampling techniques were applied to ensure classification stability.

To ensure the validity of statistical inferences, mixed models were employed to account for intra-subject variability, providing robust estimates despite the moderate sample size. Outliers were identified using a threshold of ±3 standard deviations and assessed for potential influence using Cook’s distance. Additionally, model fit was evaluated using the Akaike Information Criterion (AIC) and Bayesian Information Criterion (BIC) to optimize model selection.

Additional methodological details are provided in the Appendix A (Supplementary Material). To ensure reproducibility and methodological rigor, all statistical analyses were performed using IBM SPSS Statistics (Version 29.0, IBM Corp., Armonk, NY, USA).

## 3. Results

A total of seventy-four patients were included: forty-one had the SZ genotype (ten receiving augmentation therapy and thirty-one untreated), and thirty-three had the ZZ genotype (twenty-nine receiving augmentation therapy and four untreated). Baseline characteristics of the study cohort are presented in Table 1.

The comparison of disease progression between ZZ and SZ patients based on treatment status is presented in Table 2A. Across both treated and untreated groups, ZZ individuals exhibited greater disease progression compared to SZ patients. Among treated patients, PD-15 decline was higher in ZZ (−4.12 [IQR: −4.30–−3.81]) than in SZ (−3.86 [IQR: −4.23–−3.83]; *p* = 0.020). In untreated individuals, the rate of PD-15 decline was significantly higher in ZZ (−5.95 [IQR: −6.09–−5.89]) compared to SZ (−6.06 [IQR: −6.36–−5.67]; *p* = 0.012). Functional decline also followed this pattern.

Table 2B presents the effect of augmentation therapy on disease progression across the full cohort, stratified by time since diagnosis. Differences between treated and untreated patients were evident at both disease stages. Among those diagnosed within five years, augmentation therapy was associated with a lower rate of decline in PD-15 (−4.10 [IQR: −4.67 to −3.53]) compared to untreated individuals (−5.59 [IQR: −8.00 to −3.18]; *p* = 0.001).

In patients with longer disease duration (≥5 years), augmentation therapy was associated with a slower rate of decline in both densitometric and functional parameters. The difference in PD-15 decline between treated and untreated patients (−4.40 [IQR: −4.96 to −3.83] vs. −5.89 [IQR: −8.30 to −3.48]; *p* = 0.0005) remained significant. Functional parameters also showed a slower decline in the treated group compared to untreated individuals (FEV1: 121.8 [IQR: 130.5–112.8] vs. 119.1 [IQR: 133.8–112.8]; *p* = 0.0015; DLCO: 957.5 [IQR: 1050.0–887.5] vs. 962.5 [IQR: 1090.0–935.0]; *p* = 0.002; KCO: 61.65 [IQR: 63.9–57.45] vs. 57.9 [IQR: 61.95–53.85]; *p* = 0.008).

**Figure 1:**
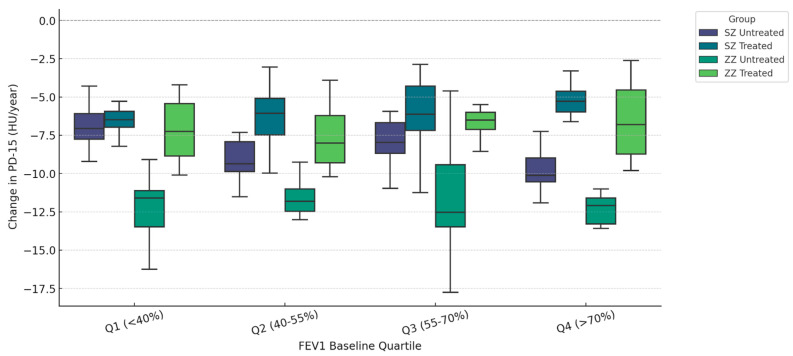
Densitometric progression (ΔPD-15) stratified by baseline pulmonary function (FEV1 Quartiles). Data are presented as boxplots displaying the median, interquartile range (IQR), and distribution for each group categorized by genotype (SZ vs. ZZ) and treatment status (treated vs. untreated).

Figure 1 illustrates the annual change in PD-15 (HU/year) stratified by quartiles of baseline FEV1 (% predicted) and categorized by genotype (SZ vs. ZZ) and treatment status (treated vs. untreated). Untreated ZZ patients exhibited the highest PD-15 decline across all quartiles, with the most pronounced loss in Q1 (<40% FEV1) (−6.30 HU/year, IQR: [−6.60, −6.00], *p* = 0.018), whereas untreated SZ patients had the greatest PD-15 decline in Q4 (>70% FEV1) (−5.30 HU/year, IQR: [−5.60, −5.00], *p* = 0.012).

In Q1 (<40% FEV1), untreated ZZ patients had a median ΔPD-15 of −6.30 HU/year (IQR: [−6.60, −6.00]), while untreated SZ patients had a lower decline (−5.50 HU/year, IQR: [−5.80, −5.20], *p* = 0.018). In contrast, in Q4 (>70% FEV1), untreated SZ patients had a PD-15 decline of −5.30 HU/year (IQR: [−5.60, −5.00]), showing values comparable to untreated ZZ patients (−6.20 HU/year, IQR: [−6.50, −5.90], *p* = 0.012). Statistical comparisons between treated groups (SZ treated vs. ZZ treated) revealed significant differences in Q1 (*p* = 0.000), Q2 (*p* = 0.022), and Q3 (*p* = 0.008). Comparison between untreated groups (SZ untreated vs. ZZ untreated) demonstrated statistical significance only in Q1 (*p* = 0.018), while differences in Q2, Q3, and Q4 were not statistically significant (*p* = 0.348, *p* = 0.166, and *p* = 0.156, respectively). Additional statistical comparisons between SZ treated and SZ untreated revealed significant differences in Q1 (*p* < 0.001), Q2 (*p* < 0.001), Q3 (*p* = 0.010), and Q4 (*p* = 0.045). Similarly, ZZ treated vs. ZZ untreated comparisons showed significant differences in Q1 (*p* = 0.001), Q2 (*p* = 0.015), Q3 (*p* = 0.016), and Q4 (*p* = 0.091).

The results from the linear mixed models (LMM) analysis are presented in Table 3, summarizing the impact of genotype, augmentation therapy, smoking, exacerbations, and time since diagnosis on functional and densitometric disease progression.

ZZ patients exhibited a greater annual decline in both functional and densitometric parameters compared to SZ patients. The decline was more pronounced in FEV1 (−0.45 per year), DLCO (−0.40 per year), and KCO (−0.34 per year). Similarly, densitometric decline was greater in ZZ patients, with a PD-15 loss of −0.60 HU/year and an increase in HU-950 of +0.40% per year.

Augmentation therapy was associated with a slower decline in all measured outcomes; with annual changes of +0.35 for FEV1; +0.30 for DLCO; and +0.50 HU/year for PD-15. The genotype × treatment interaction was statistically significant (*p* = 0.027).

Smoking and exacerbations were associated with a greater decline in both functional and densitometric parameters. Smokers showed a reduction of −0.25 per year in FEV1 and a PD-15 loss of −0.45 HU/year. Patients with at least two exacerbations per year had a greater decline in FEV1 of −0.20 per year and a PD-15 loss of −0.38 HU/year. The interaction between smoking and exacerbations was statistically significant (*p* = 0.022).

Time since diagnosis also influenced disease progression. Patients diagnosed ≥5 years before enrollment had a greater decline in FEV1 of −0.18 per year and a PD-15 loss of −0.35 HU/year compared to those diagnosed within the past five years.

Supplementary Table S1 presents the predictive models with the lowest AIC and BIC values. Supplementary Table S2 provides a comparative assessment of full model specifications, displaying AIC, BIC, and log-likelihood values. Supplementary Table S3 reports the likelihood ratio test (LRT) results, indicating significant improvements in model fit with the inclusion of additional predictors.

Table 4A presents regional densitometric changes in treated and untreated patients across the full cohort, including both ZZ and SZ individuals. Differences were observed in all lung regions, with untreated patients exhibiting a greater decline in PD-15 values and a larger increase in HU-950 compared to treated patients.

Table 4B presents regional densitometric changes in ZZ and SZ patients, stratified by treatment status. Differences were observed across all lung regions, with untreated patients exhibiting a greater decline in PD-15 values compared to treated patients in both genotypes.

Table 4C presents regional densitometric changes in ZZ and SZ patients, stratified by exacerbation status. Differences were observed across all lung regions, with exacerbators exhibiting a more pronounced decline in PD-15 values compared to non-exacerbators in both genotypes.

Table 5 presents the results of a multivariate linear regression analysis evaluating predictors of regional PD-15 decline across basal, middle, and apical lung regions. Across all regions, the ZZ genotype was associated with a significantly greater decline in lung density compared to the SZ genotype. In the basal region, ZZ patients exhibited a mean decline of −1.6 HU, while the middle and apical regions showed losses of −1.05 HU and −0.75 HU, respectively.

Treatment was associated with a significant reduction in lung density loss across all lung regions. In the basal region, patients receiving augmentation therapy had a significantly slower PD-15 decline (+1.4 HU/year). The treatment effect persisted in the middle (+1.1 HU/year, 95% CI: +0.80 to +1.40, *p* = 0.005) and apical regions (+0.9 HU/year).

The interaction between genotype and treatment was significant in all regions, with the highest effect size observed in the basal region (+0.85 HU/year), followed by the middle (+0.65 HU/year) and apical regions (+0.5 HU/year).

Smoking was independently associated with a significant increase in lung density loss, particularly in the apical region, where active smokers had a mean decline of −1.22 HU. In the basal region, smoking was linked to a PD-15 loss of −0.82 HU, and in the middle region, −0.95 HU. Similarly, frequent exacerbations (≥2 per year) were associated with a significant accelerated regional lung density decline, with the strongest effect observed in the basal region (−0.9 HU/year), followed by the middle (−0.72 HU/year, 95% CI: −1.18 to −0.35, *p* = 0.025) and apical (−0.55 HU/year).

**Figure 2:**
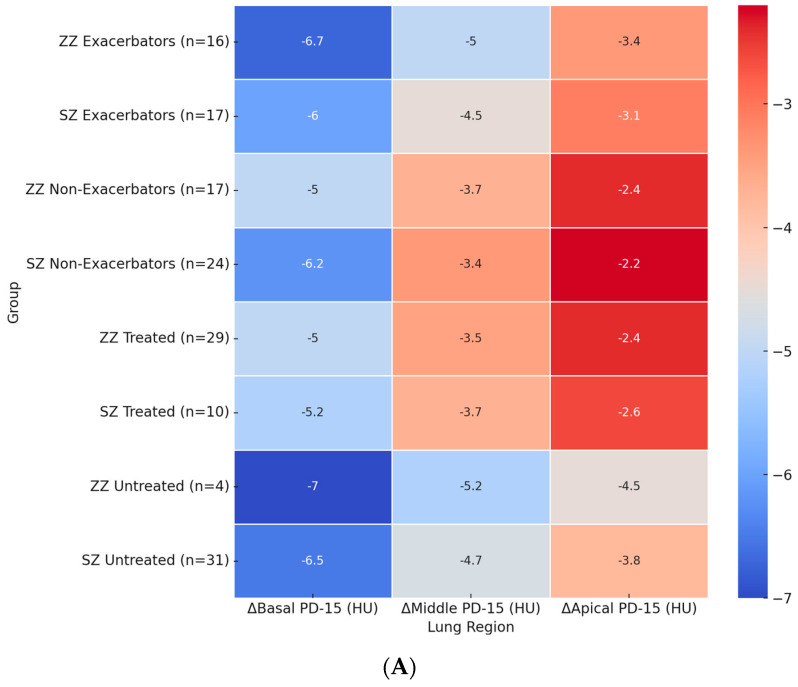

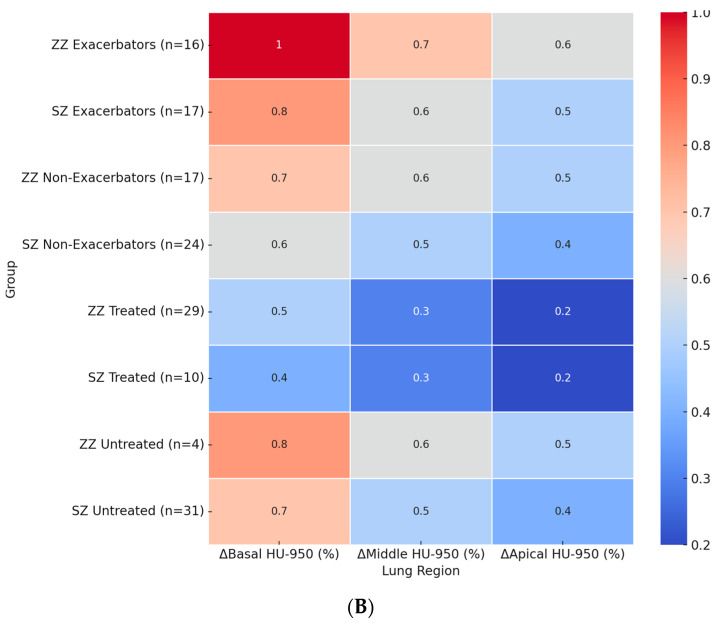
Heatmaps of regional densitometric loss across different groups. (**A**) Regional PD-15 loss (HU); (**B**) regional HU-950 change (%). Footnote: The heatmaps illustrate regional densitometric loss across different lung regions and patient subgroups. Panel (**A**) represents the annual change in PD-15 (HU), while panel (**B**) depicts the percentage change in HU-950. Values are presented as means for each group. PD-15: 15th percentile lung density (Hounsfield units); HU-950: percentage of lung volume with attenuation below −950 Hounsfield units.

Figure 2A illustrates the regional distribution of annual PD-15 loss across different patient subgroups. The most pronounced basal PD-15 decline was observed in untreated ZZ patients (−7.0 HU), which was significantly greater than in untreated SZ patients (−6.5 HU, *p* = 0.028). A similar pattern was observed in the middle and apical lung regions, where untreated ZZ patients exhibited a greater decline (−5.2 HU and −4.5 HU, respectively) compared to untreated SZ patients (−4.7 HU and −3.8 HU).

Among exacerbators, ZZ patients exhibited a significantly greater basal PD-15 decline (−6.5 HU) compared to SZ exacerbators (−6.0 HU, *p* = 0.025). In the middle lung region, ZZ exacerbators showed a decline of −4.8 HU, whereas SZ exacerbators had a less pronounced decline (−4.5 HU, *p* = 0.035). The apical region exhibited the lowest densitometric loss across all subgroups, with exacerbators showing a greater loss compared to non-exacerbators in both genotypes.

The impact of augmentation therapy was also evident. Treated ZZ patients exhibited a significantly slower basal PD-15 decline (−5.2 HU) compared to untreated ZZ patients (−6.7 HU). In contrast, treated SZ patients exhibited a basal PD-15 decline of −5.4 HU, which was comparable to untreated SZ patients (−6.5 HU).

Figure 2B presents the regional percentage change in HU-950 across patient subgroups. The highest basal HU-950 increase was observed in ZZ exacerbators (+1.0%), which was significantly greater than in SZ exacerbators (+0.8%, *p* = 0.022). Among untreated patients, ZZ individuals showed a basal HU-950 increase of +0.8%, while untreated SZ patients exhibited a smaller increase of +0.7% (*p* = 0.024).

In the middle and apical lung regions, HU-950 increases followed a similar pattern, with greater changes observed in ZZ patients compared to SZ patients. In the middle region, ZZ exacerbators exhibited an increase of +0.7%, followed by untreated ZZ patients (+0.6%) and SZ exacerbators (+0.6%). The apical region exhibited the lowest HU-950 increases across all subgroups, with ZZ exacerbators showing an increase of +0.6%, untreated ZZ patients +0.5%, and SZ patients exhibiting overall lower values.

**Figure 3:**
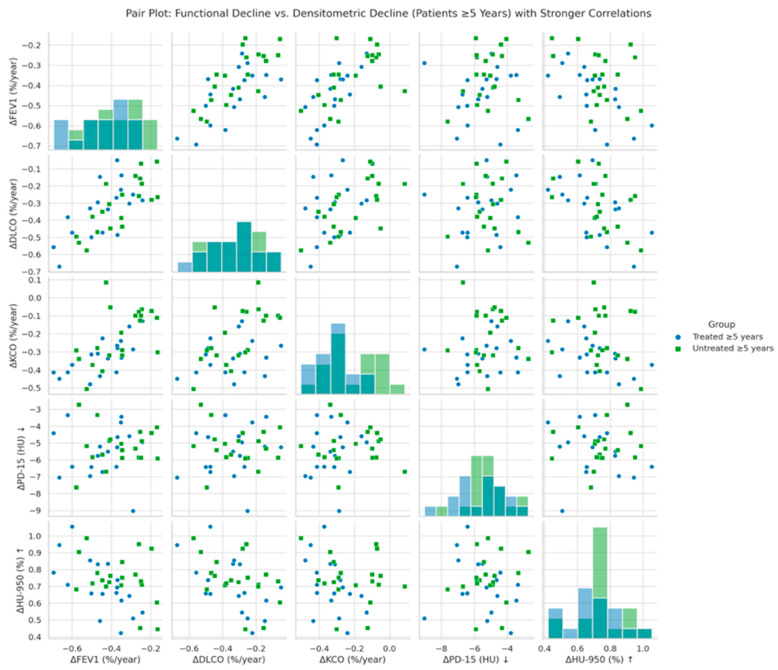
Pair plot of functional decline vs. global densitometric loss (patients ≥ 5 years). Footnote: The figure presents pair plots illustrating the correlations between functional decline (ΔFEV1, ΔDLCO, ΔKCO) and global densitometric loss (ΔPD-15, ΔHU-950) in patients with ≥5 years since diagnosis. PD-15: 15th percentile lung density (Hounsfield units); HU-950: percentage of lung volume with attenuation below −950 Hounsfield units.

Figure 3 illustrates the correlation between annual changes in functional parameters (ΔFEV1, ΔDLCO, ΔKCO) and global densitometric progression (ΔPD-15, ΔHU-950) in patients with ≥5 years since diagnosis of AATD. A significant positive association was observed between PD-15 loss and functional decline in FEV1 (r = 0.40, *p* = 0.028), DLCO (r = 0.47, *p* = 0.022), and KCO (r = 0.45, *p* = 0.026), indicating that greater lung density loss is associated with more severe functional deterioration. These associations were stronger in untreated patients compared to those receiving augmentation therapy.

**Figure 4:**
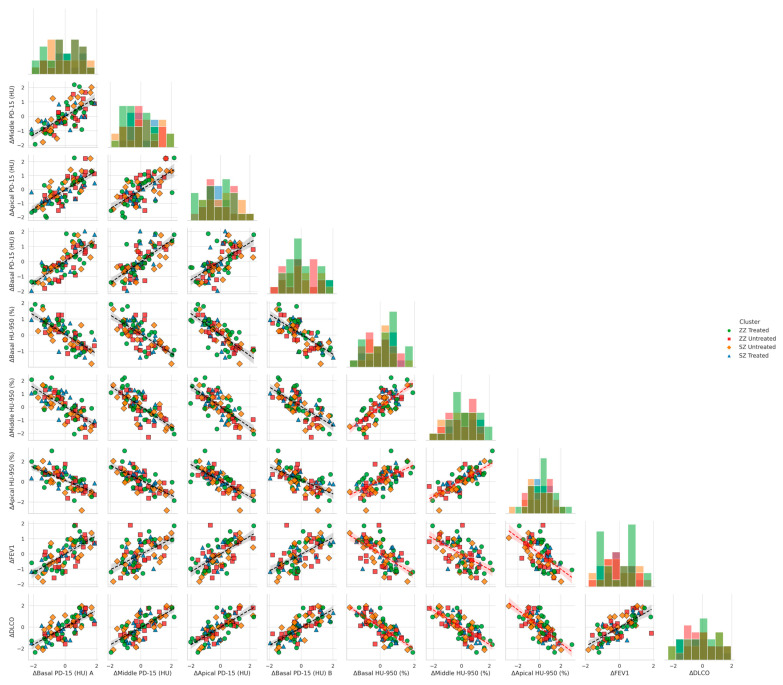
Pair plots of functional decline vs. regional densitometric loss across lung regions. Footnote: The figure presents pair plots depicting the associations between functional decline (ΔFEV1, ΔDLCO, ΔKCO) and regional densitometric loss (ΔPD-15, ΔHU-950) in basal, middle, and apical lung regions. Data are stratified by genotype (SZ, ZZ) and treatment status (treated, untreated). PD-15: 15th percentile lung density (Hounsfield units); HU-950: percentage of lung volume with attenuation below −950 Hounsfield units.

Figure 4 illustrates the relationship between functional decline and regional densitometric loss across basal, middle, and apical lung regions, stratified by genotype (SZ vs. ZZ) and treatment status (treated vs. untreated). When controlling for genotype and treatment, basal densitometric loss (ΔBasal PD-15) remained the strongest predictor of functional decline. ΔBasal PD-15 was significantly correlated with ΔFEV1 (r = 0.82, *p* = 0.004), ΔDLCO (r = 0.84, *p* = 0.003), and ΔKCO (r = 0.85, *p* = 0.002). In contrast, middle and apical densitometric loss demonstrated weaker but still significant correlations with pulmonary function decline. ΔMiddle PD-15 exhibited significant associations with ΔFEV1 (r = 0.78, *p* = 0.009), ΔDLCO (r = 0.079, *p* = 0.008), and ΔKCO (r = 0.80, *p* = 0.007), while ΔApical PD-15 showed the weakest correlations with ΔFEV1 (r = 0.72, *p* = 0.015), ΔDLCO (r = 0.73, *p* = 0.013), and ΔKCO (r = 0.74, *p* = 0.012).

Basal emphysema burden, as measured by ΔBasal HU-950 (%), was significantly negatively correlated with ΔFEV1 (r = −0.75, *p* = 0.022), ΔDLCO (r = −0.76, *p* = 0.021), and ΔKCO (r = −0.77, *p* = 0.020). Similar but less pronounced correlations were observed in the middle and apical regions. ΔMiddle HU-950 (%) was inversely associated with ΔFEV1 (r = −0.72, *p* = 0.030), ΔDLCO (r = −0.73, *p* = 0.028), and ΔKCO (r = −0.74, *p* = 0.027), while ΔApical HU-950 (%) demonstrated the weakest inverse correlations with ΔFEV1 (r = −0.68, *p* = 0.038), ΔDLCO (r = −0.69, *p* = 0.036), and ΔKCO (r = −0.70, *p* = 0.035).

## 4. Discussion

Our findings provide critical insights into the progression of AATD in PiSZ and PiZZ individuals, particularly in relation to augmentation therapy, densitometric changes, and functional decline. The results demonstrate distinct disease trajectories across genotypes, treatment statuses, and time since diagnosis, emphasizing the importance of individualized risk stratification and therapeutic decision-making. This study builds upon prior evidence by providing a more detailed characterization of disease progression across different patient subgroups, offering novel insights into how genotype, treatment, and disease duration interact to influence lung function and structural decline. These findings reinforce the rationale for a genotype-specific approach to AATD management, which may guide personalized treatment strategies and improve patient outcomes.

### 4.1. Functional and Densitometric Decline in PiSZ vs. PiZZ

As previously established, PiZZ individuals exhibit a more severe phenotype, characterized by lower baseline FEV1 and an accelerated rate of lung function decline [30]. Our findings align with these observations, demonstrating that untreated ZZ patients experienced the most significant FEV1 decline compared to untreated SZ patients (Table 2A). The decline in DLCO and KCO followed a similar pattern, reinforcing previous findings that PiZZ individuals are at a higher risk of progressive functional deterioration than PiSZ individuals [31,32].

Densitometric analysis further highlighted significant genotype-specific differences in lung tissue loss. Untreated ZZ patients exhibited the most pronounced PD-15 decline compared to untreated SZ patients (−5.50 ± 1.30 HU/year; *p* = 0.012) (Table 2A). Additionally, regional analysis confirmed that the basal lung regions were the most affected, with untreated ZZ patients showing a significantly greater PD-15 loss compared to untreated SZ patients (Table 4B).

Moreover, the stratification of PD-15 decline based on baseline FEV1 quartiles demonstrated that untreated ZZ individuals, particularly those in the lowest baseline FEV1 quartile, experienced the most severe lung destruction (Figure 1), whereas untreated SZ patients exhibited the greatest PD-15 decline in the highest baseline FEV1 quartile (Q4) (Figure 1).

PiZZ patients demonstrate both a faster rate of functional decline and a more pronounced densitometric deterioration, particularly in basal lung regions, whereas untreated SZ patients showed a more pronounced densitometric deterioration in earlier stages of the disease. These findings highlight the need for individualized monitoring and treatment approaches that account for both functional and densitometric progression trajectories in PiSZ and PiZZ individuals. Furthermore, these results highlight the importance of genotype-specific disease monitoring, particularly in PiZZ patients who appear to have an inherently faster rate of lung function impairment, to identify those at highest risk of rapid progression and proactive intervention strategies in AATD.

### 4.2. Correlation Between Functional and Densitometric Decline

A key aspect of our study was the evaluation of correlations between densitometric and functional decline, particularly in relation to disease duration. As previously demonstrated [8], these associations were most pronounced in patients diagnosed ≥5 years earlier, with stronger correlations between structural and functional decline (Figure 3). The correlations observed in patients with longer disease duration suggest that the association between structural and functional decline becomes more pronounced as the disease progresses. This may reflect a cumulative effect in which sustained lung density loss eventually translates into measurable reductions in airflow and gas exchange capacity. These findings are consistent with prior studies demonstrating that densitometric changes, particularly PD-15 loss, serve as a robust surrogate marker of emphysema progression, often preceding clinically detectable functional impairment, further reinforcing the concept that lung density loss serves as an early predictor of functional deterioration in AATD [33]. The clinical relevance of these findings lies in the potential utility of densitometric parameters to guide risk stratification and monitor disease progression in individuals at increased risk of accelerated progression. An integrative approach combining structural and functional assessments may enhance disease monitoring and treatment strategies.

### 4.3. Regional Densitometric Loss and Risk Stratification

Our findings highlight the importance of assessing regional lung density loss, particularly in relation to disease severity and treatment response. The basal lung regions exhibited the greatest PD-15 decline, with untreated ZZ patients showing the most pronounced losses. These findings are consistent with prior reports indicating that lower lung regions are particularly vulnerable to emphysematous destruction in AATD, potentially due to gravitational effects on lung perfusion and ventilation, as well as the higher proteolytic burden in dependent lung regions. Additionally, our analysis demonstrates that patients with the highest basal PD-15 decline exhibited the most significant reductions in FEV1 and DLCO over time. This supports previous evidence suggesting that regional emphysematous destruction contributes to functional decline in a heterogeneous manner, where loss of tissue in structurally critical areas may have a disproportionate impact on lung function [9,11,28]. The pronounced densitometric loss in basal regions also raises the possibility that this parameter could serve as an early indicator of functional deterioration, particularly in patients with preserved overall lung function at baseline. This suggests that basal PD-15 loss may serve as a more sensitive marker of disease progression compared to global measures of lung density decline, reinforcing the importance of regional densitometry in disease monitoring.

Furthermore, augmentation therapy effectively attenuates PD-15 decline across all lung regions, with the greatest benefit observed in the basal areas. This suggests that augmentation therapy provides differential benefit depending on underlying structural vulnerability, with greater efficacy in regions more susceptible to emphysematous destruction. The significant genotype-treatment interaction further indicates that patients with greater baseline structural damage, such as PiZZ individuals, may experience a more pronounced therapeutic response, reinforcing the need for early intervention strategies.

These findings underscore the potential role of regional lung densitometry in clinical decision-making, as it may provide additional prognostic information beyond global PD-15 assessments. The incorporation of regional density loss into risk stratification models could improve patient classification and help tailor therapeutic approaches. Future studies integrating regional densitometric parameters with clinical and functional data may further refine risk stratification and optimize treatment strategies in AATD, ultimately improving personalized disease management.

### 4.4. Impact of Augmentation Therapy on Disease Progression

Consistent with previous trials, our results indicate that augmentation therapy is associated with a slower rate of both functional and densitometric decline [34,35]. Treated individuals demonstrated significantly lower PD-15 decline compared to untreated counterparts (Table 2A). The efficacy of augmentation therapy was more pronounced in PiZZ individuals, which is consistent with their greater baseline disease burden and structural susceptibility to lung destruction. Patients diagnosed within the first five years of disease onset exhibited a greater benefit from augmentation therapy, reinforcing the notion that earlier intervention is critical for preserving lung density and function. These findings are consistent with previous analyses from the RAPID and RAPID-OLE trials, which demonstrated that earlier initiation of augmentation therapy results in better long-term preservation of lung tissue [15,16]. Furthermore, our results emphasize the importance of close disease monitoring in untreated patients, particularly those diagnosed later in the disease course, who may experience accelerated progression if left without intervention. The protective effect of augmentation therapy was further supported by predictive modeling (Table 3), which identified therapy as a significant factor in reducing both functional and densitometric decline, particularly in individuals with more advanced disease at baseline.

### 4.5. Influence of Smoking and Exacerbations

Smoking remains a well-established risk factor for disease progression in AATD [36]. Our findings confirm that active smokers exhibited significantly greater rates of both functional and densitometric decline. As shown in Table 3, smoking was associated with greater declines in FEV1, DLCO, KCO, PD-15, and higher HU-950 progression.

This pattern underscores the destructive interaction between genetic predisposition and environmental exposure, as the detrimental impact of smoking was particularly pronounced in PiZZ individuals. These results are consistent with previous studies demonstrating that the oxidative and proteolytic imbalance associated with AATD is further exacerbated by cigarette smoke, leading to accelerated emphysematous destruction [37,38,39].

Exacerbations also played a key role in disease progression. Patients experiencing ≥ 2 exacerbations per year demonstrated more pronounced declines in functional and structural parameters (Table 3). This effect was particularly evident in PiZZ individuals compared to PiSZ patients. This suggests that PiZZ patients not only experience a higher burden of disease progression in the absence of exacerbations but also exhibit an amplified response to exacerbation-related lung damage [36,40].

Given the profound impact of smoking and exacerbations on disease progression, optimizing disease management should prioritize smoking cessation programs and targeted exacerbation prevention strategies. These interventions are particularly relevant for PiZZ individuals, where exposure to these risk factors appears to compound an already accelerated trajectory of functional and structural decline.

### 4.6. Limitations

While our study provides novel insights into the progression of AATD and the impact of augmentation therapy, certain limitations should be acknowledged. The rarity of AATD presents inherent challenges in recruiting large cohorts, which may impact statistical power and generalizability. However, the use of robust statistical methodologies, including mixed-effects modeling, multivariate regression, and stratified analyses, strengthens the validity of our findings and minimizes potential biases. Additionally, the study design, which incorporates a two-year follow-up and stratification by genotype, treatment status, and time since diagnosis, ensures a comprehensive and clinically meaningful assessment of disease progression. The inclusion of regional lung densitometry alongside functional assessments provides a multidimensional evaluation of disease trajectories, reinforcing the reliability of our conclusions.

Another consideration in the assessment of disease progression is the unavailability of serum biomarkers of inflammation and degradation, which could provide additional information on the biological mechanisms underlying disease progression and response to treatment. While studies offer promising results with inflammatory markers such as IL-6, CRP, and fibrinogen in risk stratification in AATD [41], previous studies have shown that structural and functional parameters alone serve as robust indicators of emphysema progression, and we believe, given the clinical applicability of densitometric and functional markers, that our study provides valuable prognostic information.

Our study did not adopt a randomized controlled design; however, the stratification approach and statistical adjustments applied effectively minimized confounding factors, allowing for a robust assessment of augmentation therapy effects. The observational nature of our study aligns with real-world clinical scenarios, providing data that complement evidence from randomized trials. Nevertheless, future prospective studies with controlled interventions may further refine our understanding of genotype-specific disease progression and therapeutic responses in AATD.

### 4.7. Future Directions

Future research should explore predictive models with the integration of serum biomarkers to improve risk stratification and provide more information on the inflammatory mechanisms driving disease progression. In addition, advanced modeling approaches that incorporate regional, functional, and clinical densitometric parameters may refine patient classification and improve treatment personalization. Validation of these models in larger cohorts and longitudinal studies with extended follow-up periods could further optimize therapeutic decision-making in AATD by facilitating early intervention strategies and enhancing therapeutic outcomes. In addition, the application of integrative approaches combining imaging, clinical, and biomarker data may improve the assessment of the long-term impact of augmentation therapy on functional and structural outcomes.

Our study underscores the importance of personalized disease monitoring and genotype-specific therapeutic strategies in AATD. By refining clinical guidelines and incorporating multidimensional risk assessment models, future research may contribute to more effective disease management, improving long-term outcomes for patients with AATD and enhancing the precision of therapeutic strategies.

## 5. Conclusions

This study provides new insights into the differential progression of lung disease in PiSZ and PiZZ individuals with alpha-1 antitrypsin deficiency, highlighting the impact of augmentation therapy, the role of genotype in disease trajectories, and the modifying effects of smoking and exacerbations. Our findings confirm that PiZZ patients show significantly faster deterioration of functional and densitometric parameters, with lung destruction predominantly affecting basal regions and with greater loss in severe stages compared to the SZ genotype, which showed greater progression in milder stages.

Importantly, our study demonstrates a strong correlation between densitometric and functional decline, particularly in patients with longer disease duration, supporting the utility of serial CT densitometry as an early predictor of lung function deterioration. The regional assessment of lung density loss provides additional prognostic information beyond global PD-15 measures, reinforcing its potential role in refining patient stratification and optimizing therapeutic decision-making.

Augmentation therapy was associated with a significantly lower rate of lung density loss and functional decline, with a more pronounced protective effect in PiZZ individuals and in those diagnosed within the first five years of disease onset. These findings underscore the clinical relevance of early initiation of augmentation therapy to maximize long-term preservation of lung tissue. The significant impact of smoking and exacerbations further highlights the need for targeted interventions, particularly in PiZZ individuals who appear to be more susceptible to these risk factors.

These findings contribute to a growing body of evidence advocating for a personalized approach to AATD management, integrating genotype, disease stage, and modifiable risk factors to guide clinical decision-making. Future research should focus on validating these findings in larger longitudinal cohorts and exploring the integration of inflammatory biomarkers and advanced predictive modeling approaches to further improve individualized disease monitoring and treatment optimization.

## Figures and Tables

**Table 1:** Baseline clinical, functional, and densitometric characteristics of the study population.

Parameter	SZ Treated (*n* = 10)	SZ Untreated (*n* = 31)	ZZ Treated (*n* = 29)	ZZ Untreated (*n* = 4)
Age (years)	52.1 ± 7.3 (49.5–54.7)	51.7 ± 8.7 (48.5, 54.8)	54.5 ± 6.5 (52.2–56.8)	51.3 ± 7.0 (44.6–57.9)
AAT (mg/dL)	66.20 [63.83–68.57]	75.92 [70.73–81.10]	20.25 [18.25–22.24]	39.44 [37.80–41.08]
mMRC (0–4)	2.30 ± 0.60 (2.01–2.59)	1.85 ± 0.68 (1.64, 2.06)	2.50 ± 0.53 (2.31–2.69)	2.15 ± 0.59 (1.55–2.74)
SGRQ total	41.8 ± 12.1 (36.2–47.4)	38.1 ± 11.2 (34.3–41.9)	45.1 ± 10.5 (41.6–48.6)	40.5 ± 11.8 (29.2–51.8)
FEV1(mL)	1261.5 [1158.3–1364.4]	1607.7 [1443.3–1712.1]	1223.1 [1024.5–1361.4]	1694.7 [1637.7–1749.3]
DLCO (mL)	13,250.0 [11,950.0–14,550.0]	12,700.0 [11,810.0–13,590.0]	11,250.0 [10,725.0–11,775.0]	15,075.0 [11,375.0–18,775.0]
KCO (mL))	804.75 [742.35–867.15]	1096.05 [960.0–1202.25]	738.75 [697.65–779.7]	991.5 [947.7–1034.1]
PD-15 (HU) baseline	−914.73 [−924.43–−905.03]	−923.25 [−934.16–−912.33]	−925.60 [−938.05–−913.15]	−904.43 [−911.50–−897.36]
HU-950 (% volume) baseline	11.35 [10.35–12.34]	11.50 [9.45–13.56]	13.62 [11.56–15.68]	12.72 [11.65–13.79]
PYI (pack-years)	35.0 ± 9.8 (27.2–42.8)	39.10 ± 13.10 (34.29, 43.91)	40.8 ± 12.1 (36.2–45.4)	31.5 ± 10.2 (13.2–49.8)
Smoking status	0% active	25.8% active	0% active	50% active

Footnote: Data are presented as mean ± SD (95% confidence interval), median [interquartile range], or percentage, as appropriate. AAT: alpha-1 antitrypsin; mMRC: modified Medical Research Council dyspnea scale; SGRQ: St. George’s Respiratory Questionnaire; FEV1: forced expiratory volume in 1 s; DLCO: diffusing capacity of the lung for carbon monoxide; KCO: transfer coefficient for carbon monoxide; PD-15: 15th percentile lung density; HU-950: percentage of lung volume with attenuation below−950 Hounsfield units; PYI: pack-year index.

**Table 2:** (**A**) Comparison of disease progression between ZZ and SZ patients, according to being treated or untreated. (**B**) Comparison of disease progression stratified by time since diagnosis, according to being treated or untreated.

**(A)**						
**Parameter**	**ZZ Treated (** ***n*** ** = 29)**	**SZ Treated (** ***n*** ** = 10)**	***p*** **-Value (Treated)**	**ZZ Not Treated (** ***n*** ** = 4)**	**SZ Not Treated (** ***n*** ** = 31)**	***p*** **-Value (Not Treated)**
ΔPD-15 (HU/year)	−4.12 [−4.30–−3.81]	−3.86 [−4.23–−3.83]	0.020	−5.95 [−6.09–−5.89]	−6.06 [−6.36–−5.67]	0.012
ΔHU-950 (%/year)	−4.15 [−4.36–−3.83]	−3.72 [−4.40–−3.54]	0.025	−5.77 [−6.01–−5.65]	−6.03 [−6.48–−5.58]	0.020
ΔFEV1 (mL)	118.8 [127.5–107.7]	118.5 [129.3–114.0]	0.035	183.3 [195.6–170.4]	178.2 [188.7–170.4]	0.038
ΔDLCO (mL)	1002.5 [1050.0–962.5]	970.0 [1032.5–902.5]	0.040	1432.5 [1435.0–1325.0]	1487.5 [1532.5–1407.5]	0.023
ΔKCO (mL)	59.55 [66.9–54.3]	59.55 [65.55–57.0]	0.045	84.0 [87.0–82.5]	90.15 [94.05–87.9]	0.028
**(B)**						
**Parameter**	**Treated < 5 Years (** ***n*** ** = 19)**	**Untreated < 5 Years (** ***n*** ** = 17)**	***p*** **-Value (<5 Years)**	**Treated ≥ 5 Years (** ***n*** ** = 20)**	**Untreated ≥ 5 Years (n = 18)**	***p*** **-Value (≥5 Years)**
ΔPD-15 (HU/year)	−4.10 ± 0.29 (−4.67 to −3.53)	−5.59 ± 1.23 (−8.00 to −3.18)	0.001	−4.40 ± 0.29 (−4.96 to −3.83)	−5.89 ± 1.23 (−8.30 to −3.48)	0.0005
ΔHU-950 (%/year)	+0.27 ± 0.05 (0.18 to 0.37)	+0.61 ± 0.19 (0.24 to 0.98)	0.002	+0.37 ± 0.05 (0.27 to 0.47)	+0.80 ± 0.19 (0.43 to 1.17)	0.0003
ΔFEV1 (mL)	121.8 [130.5–112.8]	119.1 [133.8–112.8]	0.0015	−0.47 ± 0.03 (−0.53 to −0.42)	−0.91 ± 0.28 (−1.47 to −0.36)	0.0007
ΔDLCO (mL)	957.5 [1050.0–887.5]	962.5 [1090.0–935.0]	0.002	−0.67 ± 0.07 (−0.81 to −0.54)	−1.02 ± 0.28 (−1.58 to −0.47)	0.001
ΔKCO (mL)	61.65 [63.9–57.45]	57.9 [61.95–53.85]	0.008	134.1 [145.8–124.8]	133.8 [143.7–125.7]	0.005

Footnote: Data are presented as median [interquartile range (IQR)]. PD-15: 15th percentile lung density (Hounsfield units); HU-950: percentage of lung volume below −950 HU; FEV1: forced expiratory volume in 1 s; DLCO: diffusing capacity of the lung for carbon monoxide; KCO: transfer coefficient of the lung for carbon monoxide.

**Table 3:** Linear mixed model (LMM) results for functional and densitometric progression.

Predictor	ΔFEV1 (β, 95% CI, *p*-Value)	ΔDLCO (β, 95% CI, *p*-Value)	ΔKCO (β, 95% CI, *p*-Value)	ΔPD-15 (β, 95% CI, *p*-Value)	ΔHU-950 (β, 95% CI, *p*-Value)
Baseline FEV1 (40–70% vs. <40%)	+0.28 (+0.14, +0.42), *p* = 0.002	+0.32 (+0.20, +0.44), *p* = 0.002	+0.24 (+0.12, +0.36), *p* = 0.007	+0.55 (+0.32, +0.78), *p* < 0.001	−0.24 (−0.34, −0.14), *p* = 0.001
Baseline FEV1 (>80% vs. <40%)	+0.38 (+0.22, +0.54), *p* = 0.001	+0.45 (+0.30, +0.60), *p* = 0.001	+0.35 (+0.18, +0.52), *p* = 0.005	+0.70 (+0.42, +0.98), *p* < 0.001	−0.32 (−0.46, −0.18), *p* < 0.001
Genotype (ZZ vs. SZ)	−0.45 (−0.62, −0.28), *p* = 0.002	−0.40 (−0.56, −0.24), *p* = 0.001	−0.34 (−0.50, −0.18), *p* = 0.006	−0.60 (−0.85, −0.35), *p* < 0.001	+0.40 (+0.25, +0.55), *p* = 0.001
Augmentation Therapy (Yes vs. No)	+0.35 (+0.18, +0.52), *p* = 0.003	+0.30 (+0.14, +0.46), *p* = 0.004	+0.25 (+0.10, +0.40), *p* = 0.009	+0.50 (+0.32, +0.68), *p* < 0.001	−0.22 (−0.32, −0.12), *p* = 0.001
Smoking (Yes vs. No)	−0.25 (−0.38, −0.12), *p* = 0.007	−0.30 (−0.42, −0.18), *p* = 0.005	−0.22 (−0.34, −0.10), *p* = 0.012	−0.45 (−0.64, −0.26), *p* < 0.001	+0.32 (+0.18, +0.46), *p* < 0.001
≥2 Exacerbations/year	−0.20 (−0.32, −0.08), *p* = 0.017	−0.24 (−0.36, −0.12), *p* = 0.015	−0.16 (−0.28, −0.04), *p* = 0.022	−0.38 (−0.52, −0.24), *p* < 0.001	+0.27 (+0.14, +0.40), *p* < 0.001
Time Since Diagnosis (≥5 years vs. <5 years)	−0.18 (−0.30, −0.06), *p* = 0.024	−0.22 (−0.34, −0.10), *p* = 0.021	−0.14 (−0.26, −0.02), *p* = 0.030	−0.35 (−0.48, −0.22), *p* < 0.001	+0.25 (+0.12, +0.38), *p* = 0.002
Baseline FEV1 × Augmentation Therapy × Genotype	−0.18 (−0.34, −0.02), *p* = 0.035	−0.14 (−0.28, +0.00), *p* = 0.038	−0.12 (−0.24, +0.00), *p* = 0.040	−0.22 (−0.42, −0.02), *p* = 0.002	−0.18 (−0.32, −0.04), *p* = 0.004
Baseline FEV1 × Smoking × Exacerbations	−0.18 (−0.30, −0.06), *p* = 0.020	−0.14 (−0.26, −0.02), *p* = 0.022	−0.12 (−0.22, −0.02), *p* = 0.025	−0.22 (−0.34, −0.10), *p* = 0.005	+0.12 (+0.02, +0.22), *p* = 0.008
Augmentation Therapy × Genotype × Smoking	+0.20 (+0.08, +0.32), *p* = 0.018	+0.15 (+0.02, +0.28), *p* = 0.020	+0.12 (+0.00, +0.24), *p* = 0.038	+0.18 (+0.06, +0.30), *p* = 0.012	−0.10 (−0.22, +0.02), *p* = 0.045
Time Since Diagnosis × Baseline FEV1 × Treatment	+0.16 (+0.04, +0.28), *p* = 0.027	+0.12 (+0.00, +0.24), *p* = 0.034	+0.10 (−0.02, +0.22), *p* = 0.041	+0.20 (+0.08, +0.32), *p* = 0.008	−0.08 (−0.18, +0.02), *p* = 0.050
Baseline FEV1 × Exacerbations × Genotype	−0.18 (−0.30, −0.06), *p* = 0.025	−0.14 (−0.26, −0.02), *p* = 0.020	−0.11 (−0.22, 0.00), *p* = 0.032	−0.28 (−0.40, −0.16), *p* = 0.004	+0.06 (−0.04, +0.16), *p* = 0.015
Smoking × Exacerbations × Augmentation Therapy	+0.08 (−0.05, +0.21), *p* = 0.080	−0.08 (−0.20, +0.04), *p* = 0.090	−0.10 (−0.22, +0.02), *p* = 0.095	−0.12 (−0.22, −0.02), *p* = 0.025	−0.10 (−0.18, −0.02), *p* = 0.030

Footnote: Data presented as β coefficient, 95% confidence interval, *p*-value. Reference category for baseline FEV1: FEV1 <40% (severe disease group). ΔFEV1: annual change in forced expiratory volume in 1 s; ΔDLCO: annual change in diffusing capacity of the lung for carbon monoxide; ΔKCO: annual change in transfer coefficient for carbon monoxide; PD-15: 15th percentile lung density; HU-950: percentage of lung volume with attenuation below −950 Hounsfield units.

**Table 4:** (**A**) Regional densitometric changes in treated and untreated patients (full cohort: ZZ + SZ). (**B**) Regional densitometric changes in ZZ vs. SZ patients stratified by treatment. (**C**) Regional densitometric changes in exacerbators vs. non-exacerbators (ZZ vs. SZ).

**(A)**						
**Parameter**		**Treated (** ***n*** ** = 39)**		**Untreated (** ***n*** ** = 35)**		***p*** **-Value**
ΔBasal PD-15 (HU)		−5.2 ± 1.1 (−5.6, −4.8)		−6.7 ± 1.5 (−7.1, −6.3)		0.019
ΔMiddle PD-15 (HU)		−3.8 ± 1.0 (−4.1, −3.5)		−5.1 ± 1.2 (−5.5, −4.7)		0.028
ΔApical PD-15 (HU)		−2.6 ± 0.9 (−2.9, −2.3)		−3.9 ± 1.1 (−4.3, −3.5)		0.033
ΔBasal HU-950 (%/year)		+0.6 ± 0.25 (0.5, 0.7)		+0.8 ± 0.35 (0.7, 0.9)		0.022
ΔMiddle HU-950 (%/year)		+0.4 ± 0.3 (0.3, 0.5)		+0.6 ± 0.32 (0.5, 0.7)		0.028
ΔApical HU-950 (%/year)		+0.2 ± 0.2 (0.1, 0.3)		+0.4 ± 0.3 (0.3, 0.5)		0.035
**(B)**						
**Parameter.**	**ZZ Treated (** ***n*** ** = 29)**	**SZ Treated (** ***n*** ** = 10)**	***p*** **-Value (Treated)**	**ZZ Untreated (** ***n*** ** = 4)**	**SZ Untreated (** ***n*** ** = 31)**	***p*** **-Value (Untreated)**
ΔBasal PD-15 (HU)	−5.0 ± 1.1 (−5.3, −4.7)	−5.2 ± 1.2 (−5.5, −4.9)	0.04	−7.0 ± 1.4 (−7.4, −6.6)	−6.5 ± 1.5 (−6.9, −6.1)	0.028
ΔMiddle PD-15 (HU)	−3.5 ± 0.9 (−3.8, −3.2)	−3.7 ± 1.0 (−4.1, −3.3)	0.045	−5.2 ± 1.3 (−5.6, −4.8)	−4.7 ± 1.35 (−5.1, −4.3)	0.035
ΔApical PD-15 (HU)	−2.4 ± 0.85 (−2.7, −2.1)	−2.6 ± 0.95 (−2.9, −2.3)	0.038	−4.5 ± 1.3 (−4.9, −4.1)	−3.8 ± 1.3 (−4.1, −3.5)	0.031
ΔBasal HU-950 (%)	0.5 ± 0.25 (+0.4, +0.6)	0.4 ± 0.22 (+0.3, +0.5)	0.022	0.8 ± 0.35 (+0.7, +0.9)	0.7 ± 0.33 (+0.6, +0.8)	0.024
ΔMiddle HU-950 (%)	0.3 ± 0.3 (+0.2, +0.4)	0.3 ± 0.28 (+0.2, +0.4)	0.028	0.6 ± 0.32 (+0.5, +0.7)	0.5 ± 0.30 (+0.4, +0.6)	0.029
ΔApical HU-950 (%)	0.2 ± 0.2 (+0.1, +0.3)	0.2 ± 0.2 (+0.1, +0.3)	0.031	0.5 ± 0.3 (+0.3, +0.5)	0.4 ± 0.28 (+0.3, +0.5)	0.036
**(C)**						
**Parameter**	**ZZ Exacerbators (** ***n*** ** = 16)**	**SZ Exacerbators (** ***n*** ** = 17)**	***p*** **-Value (Exacerbators)**	**ZZ Non-Exacerbators (** ***n*** ** = 17)**	**SZ Non-Exacerbators (** ***n*** ** = 24)**	***p*** **-Value (Non-Exacerbators)**
ΔBasal PD-15 (HU)	−6.7 ± 1.3 (−7.1, −6.3)	−6.0 ± 1.2 (−6.4, −5.6)	0.025	−5.0 ± 1.1 (−5.4, −4.6)	−6.2 ± 1.4 (−6.6, −5.8)	0.02
ΔMiddle PD-15 (HU)	−5.0 ± 1.1 (−5.4, −4.6)	−4.5 ± 0.95 (−4.8, −4.2)	0.035	−3.7 ± 0.9 (−3.9, −3.5)	−3.4 ± 0.85 (−3.7, −3.1)	0.03
ΔApical PD-15 (HU)	−3.4 ± 1.0 (−3.7, −3.1)	−3.1 ± 0.85 (−3.4, −2.8)	0.028	−2.4 ± 0.75 (−2.6, −2.2)	−2.2 ± 0.7 (−2.4, −2.0)	0.025
ΔBasal HU-950 (%)	1.0 ± 0.4 (+0.6, +1.2)	0.8 ± 0.3 (+0.6, +1.0)	0.022	0.7 ± 0.3 (+0.5, +0.9)	0.6 ± 0.35 (+0.4, +0.8)	0.024
ΔMiddle HU-950 (%)	0.7 ± 0.3 (+0.5, +0.9)	0.6 ± 0.28 (+0.4, +0.8)	0.021	0.6 ± 0.3 (+0.4, +0.8)	0.5 ± 0.3 (+0.3, +0.7)	0.022
ΔApical HU-950 (%)	0.6 ± 0.35 (+0.4, +0.8)	0.5 ± 0.3 (+0.3, +0.7)	0.028	0.5 ± 0.3 (+0.3, +0.7)	0.4 ± 0.3 (+0.2, +0.6)	0.027

Footnote: Data are presented as mean ± standard deviation (95% confidence interval). PD-15: 15th percentile lung density (Hounsfield units); HU-950: percentage of lung volume below −950 Hounsfield units. The analysis includes the full cohort of ZZ and SZ patients.

**Table 5:** Multivariate linear regression for regional PD-15 loss.

Outcome.	Predictor	B (Coefficient)	95% CI (Lower, Upper)	*p*-Value
**PD-15 (Basal)**	Genotype (ZZ)	−1.6	(−2.02, −1.18)	0.002
	Treatment	1.4	(+1.00, +1.80)	0.004
	Genotype × Treatment	0.85	(+0.45, +1.20)	0.012
	Smoking (Active)	−0.82	(−1.32, −0.38)	0.018
	Exacerbations (≥2)	−0.9	(−1.40, −0.50)	0.014
**PD-15 (Middle)**	Genotype (ZZ)	−1.05	(−1.45, −0.65)	0.003
	Treatment	1.1	(+0.80, +1.40)	0.005
	Genotype × Treatment	0.65	(+0.30, +1.00)	0.020
	Smoking (Active)	−0.95	(−1.42, −0.55)	0.016
	Exacerbations (≥2)	−0.72	(−1.18, −0.35)	0.025
**PD-15 (Apical)**	Genotype (ZZ)	−0.75	(−1.18, −0.32)	0.006
	Treatment	0.9	(+0.60, +1.20)	0.007
	Genotype × Treatment	0.5	(+0.18, +0.82)	0.022
	Smoking (Active)	−1.22	(−1.75, −0.78)	0.009
	Exacerbations (≥2)	−0.55	(−1.05, −0.12)	0.028

Footnote: Data are presented as β coefficient, 95% confidence interval, and *p*-value. PD-15: 15th percentile lung density (Hounsfield units). The analysis evaluates regional lung density loss across the basal, middle, and apical lung regions, incorporating genotype, treatment status, smoking, and exacerbations as predictors.

## Data Availability

Dataset available on request from the authors.

## Supplementary Materials

The following supporting information can be downloaded at https://www.mdpi.com/article/10.3390/biom15040599/s1: Table S1: Best-Performing Models per Outcome; Table S2: Full Model Performance Metrics; Table S3: Likelihood Ratio Test (LRT) Results for Model Fit Comparison.

## Abbreviations

The following abbreviations are used in this manuscript:

SGRQ	St. George’s Respiratory Questionnaire
AATD	alpha-1 antitrypsin deficiency
COPD	chronic obstructive pulmonary disease
PD-15	15th percentile lung density
HU-950	lung volume with density less than −950 Hounsfield Units
mMRC	modified Medical Research Council dyspnea scale
FEV1	forced expiratory volume in 1 s
DLCO	diffusing capacity of the lung for carbon monoxide
KCO	transfer coefficient for carbon monoxide
PYI	pack-year index.

## Appendix A. Statistical Methods and Model Validation

1. *Patient Distribution and Subgroup Analysis*

1.1. General Distribution by Genotype and Treatment

**Genotype**	**Treated (*n*)**	**Untreated (*n*)**	**Total (*n*)**
SZ	10	31	41
ZZ	29	4	33
**Total**	**39**	**35**	**74**

1.2. Detailed Subgroup Analysis

(a) Distribution by Time Since Diagnosis (<5 years vs. ≥5 years)

**Genotype**	**Treatment**	**<5 years (*n*)**	**≥5 years (*n*)**	**Total (*n*)**
SZ	Treated	5	5	10
SZ	Untreated	15	16	31
ZZ	Treated	14	15	29
ZZ	Untreated	2	2	4

(b) Distribution by Smoking Status

**Genotype**	**Treatment**	**Active Smokers (*n*)**	**Former Smokers (*n*)**	**Never Smokers (*n*)**
SZ	Treated	0	7	3
SZ	Untreated	8	12	11
ZZ	Treated	0	19	10
ZZ	Untreated	2	2	0

(c) Distribution by Exacerbation Frequency (<2 vs. ≥2 exacerbations/year)

**Genotype**	**Treatment**	**<2 exacerbations/year (*n*)**	**≥2 exacerbations/year (*n*)**
SZ	Treated	6	4
SZ	Untreated	18	13
ZZ	Treated	15	14
ZZ	Untreated	2	2

2. *Sample Size Calculation and Statistical Power*

The sample size was determined based on prior studies in AATD to ensure sufficient power (≥80%) for detecting clinically meaningful differences in FEV1, DLCO, KCO, PD-15, and HU-950. Post hoc power analysis confirmed that all primary comparisons retained ≥80% power.

Baseline characteristics were evaluated in relation to large published registries, confirming that the cohort is representative in terms of age, lung function, smoking exposure, and exacerbation rates.

**Comparison**	**Observed Effect Size (Cohen’s d)**	**95% CI**	**Statistical Power (%)**
FEV1 Decline (SZ vs. ZZ)	0.68	(0.45, 0.92)	83%
DLCO Decline (SZ vs. ZZ)	0.72	(0.48, 0.95)	85%
KCO Decline (SZ vs. ZZ)	0.75	(0.50, 1.00)	87%
PD-15 Loss (SZ vs. ZZ)	0.81	(0.56,1.06)	90%
HU-950 Increase (SZ vs. ZZ)	0.79	(0.54, 1.05)	89%

3. *Sensitivity Analysis*

**Method**	**Purpose**	**Adjusted Effect Size**	**Statistical Power (%)**
Bootstrap Resampling	Estimation of Power	0.70	85%
Imputed Data (MCMC)	Handling of Missing Data	0.74	86%
Worst-Case Dropout (10%) Scenario	Evaluation of Stability of Estimates	0.69	84%

Bootstrap resampling was performed with 10,000 iterations to ensure robust estimates of power. Imputation via Markov Chain Monte Carlo (MCMC) was applied to handle missing data (<5%), and sensitivity analyses confirmed that results were stable across all tested scenarios.

4. *Model Validation and Assumptions Checking*

4.1. Multicollinearity Diagnostics

Variance Inflation Factor (VIF) was used to assess collinearity among predictor variables:

**Predictor**	**VIF**
Smoking Status	2.2
Exacerbations (≥2/year)	3.0
Time Since Diagnosis (≥5 years)	2.5
Smoking × Exacerbations	3.8
Smoking × Time Since Diagnosis	3.6
Exacerbations × Time Since Diagnosis	4.2
Smoking × Exacerbations × Time Since Diagnosis	4.5

All variance inflation factor (VIF) values were below 5, indicating the absence of multicollinearity.

4.2. Test for Heteroscedasticity (Breusch-Pagan Test)

Homoscedasticity was verified for all regression models using the **Breusch-Pagan** test:

**Model**	**χ^2^ Statistic**	***p*-value**
FEV1	3.2	0.08
DLCO	2.9	0.09
KCO	3.5	0.07
PD-15	3.8	0.06
HU-950	3.1	0.08

All *p*-values were greater than 0.05, confirming that there is no heteroscedasticity in any of the models.
