# Supplementary material for: Progression and Augmentation Therapy in PiSZ and PiZZ Alpha-1 Antitrypsin Deficiency: A Longitudinal Functional and Densitometric Study
Source: Biomolecules. 2025 Apr 17;15(4):599. doi: 10.3390/biom15040599 (PMC12024921; doi:10.3390/biom15040599)
Supplement: Supplementary file 1 [file biomolecules-15-00599-s001.zip › Supplementary tables .pdf]

Supplementary Table S1. Best-Performing Models per Outcome

Outcome	Best Model	AIC	BIC	Log-Likelihood
ΔFEV1	Baseline FEV1 + Genotype + Treatment	320	340	-150
ΔDLCO	Baseline FEV1 + Genotype + Treatment	280	300	-140
ΔKCO	Baseline FEV1 + Genotype + Treatment	290	310	-145
ΔPD-15	Baseline FEV1 + Genotype + Treatment	310	330	-148
ΔHU-950	Baseline FEV1 + Genotype + Treatment	300	320	-142

Footnote: Models incorporate different combinations of baseline FEV1, genotype, treatment status, smoking, exacerbations, and their interactions to assess their impact on disease progression. Lower AIC and BIC values indicate better model fit, while higher Log-Likelihood values reflect stronger explanatory power. Abbreviations: AIC, Akaike Information Criterion; BIC, Bayesian Information Criterion; Log-Likelihood, measure of model fit; FEV1, forced expiratory volume in one second; DLCO, diffusing capacity of the lung for carbon monoxide; KCO, transfer coefficient of the lung for carbon monoxide; PD-15, 15th percentile lung density (Hounsfield units); HU-950, percentage of lung volume with attenuation below -950 HU.

Supplementary Table S2. Full Model Performance Metrics

Outcome	Full Model	AIC	BIC	Log-Likelihood
ΔFEV1	Baseline FEV1 + Genotype + Treatment + Smoking + Exacerbations + Time Since Diagnosis	285	305	-135
ΔDLCO	Baseline FEV1 + Genotype + Treatment + Smoking + Exacerbations + Time Since Diagnosis	255	275	-128
ΔKCO	Baseline FEV1 + Genotype + Treatment + Smoking + Exacerbations + Time Since Diagnosis	265	285	-132
ΔPD-15	Baseline FEV1 + Genotype + Treatment + Smoking + Exacerbations + Time Since Diagnosis	275	295	-134
ΔHU-950	Baseline FEV1 + Genotype + Treatment + Smoking + Exacerbations + Time Since Diagnosis	265	285	-130

Footnote: Model performance was evaluated using Akaike Information Criterion (AIC), Bayesian Information Criterion (BIC), and Log-Likelihood. Lower AIC and BIC values indicate better model fit by optimizing the balance between explanatory power and model complexity. Higher Log-Likelihood values reflect stronger explanatory capacity. The full models include baseline FEV1, genotype, treatment status, smoking, exacerbations, time since diagnosis, and their interactions. Abbreviations: AIC, Akaike Information Criterion; BIC, Bayesian Information Criterion; Log-Likelihood, measure of model fit; ΔFEV1, annual change in forced expiratory volume in one second; ΔDLCO, annual change in diffusing capacity of the lung for carbon monoxide; ΔKCO, annual change in carbon monoxide transfer coefficient; ΔPD-15, annual change in 15th percentile lung density (Hounsfield units); ΔHU-950, annual change in the percentage of lung volume with attenuation below -950 HU.

Supplementary Table S3. Likelihood Ratio Test (LRT) Results for Model Fit Comparison

Outcome	LRT Statistic	p-value
**ΔFEV1**	28	0.002
**ΔDLCO**	22	0.007
**ΔKCO**	24.5	0.004
**ΔPD-15**	19.8	0.012
**ΔHU-950**	21.6	0.008

Footnote: The Likelihood Ratio Test (LRT) evaluates whether adding predictors improves model fit. Higher LRT statistics indicate greater improvement, with statistical significance assessed by p-values. Data are presented as LRT statistic and p-value. Abbreviations: FEV1, forced expiratory volume in one second; DLCO, diffusing capacity of the lung for carbon monoxide; KCO, transfer coefficient of the lung for carbon monoxide; PD-15, annual change in 15th percentile lung density (Hounsfield units); HU-950, annual change in percentage of lung volume with attenuation below -950 HU.
